# Can GPT-5.0 Interpret Thyroid Ultrasound Images? A Comparative TI-RADS Analysis with an Expert Radiologist

**DOI:** 10.3390/diagnostics16020313

**Published:** 2026-01-19

**Authors:** Yunus Yasar, Sevde Nur Emir, Muhammet Rasit Er, Mustafa Demir

**Affiliations:** Department of Radiology, Umraniye Training and Research Hospital, University of Health Sciences, 34668 Istanbul, Turkey; drsevdenuremir@gmail.com (S.N.E.); muhammeder001@gmail.com (M.R.E.); drmstfdmr1@gmail.com (M.D.)

**Keywords:** thyroid ultrasound, TI-RADS, large language models, GPT-5.0, artificial intelligence

## Abstract

**Background/Objectives:** Multimodal large language models (LLMs) may directly interpret medical images, including thyroid ultrasounds (USs). Whether these models can reliably assess thyroid nodules—where subtle echogenic and morphological details are critical—remains uncertain. The American College of Radiology (ACR) TI-RADS system provides a structured framework for benchmarking artificial intelligence. This study evaluates GPT-5.0’s ability to interpret thyroid US images according to TI-RADS criteria and contextualizes its performance relative to expert radiologist assessment, using FNA cytology as the reference standard. **Methods:** This retrospective study included 100 patients (mean age 49.8 ± 12.6 years; 72 women) with cytology-confirmed diagnoses: Bethesda II (benign) or Bethesda V–VI (malignant). Each nodule had longitudinal and transverse US images acquired with high-frequency linear probes. A board-certified radiologist (>10 years’ experience) and GPT-5.0 independently assessed TI-RADS features (composition, echogenicity, shape, margin, echogenic foci) and assigned final categories. Agreement was analyzed using Cohen’s κ, and diagnostic performance was calculated using TR4–TR5 as positive for malignancy. **Results:** Agreement was substantial for composition (κ = 0.62), shape (κ = 0.70), and margin (κ = 0.68); moderate for echogenicity (κ = 0.48); and poor for echogenic foci (κ = 0.12). GPT-5.0 demonstrated a systematic, risk-averse tendency to up-classify nodules, leading to increased TR4–TR5 assignments. Overall, the TI-RADS agreement was 58% (κ = 0.31). The radiologist showed superior diagnostic performance (sensitivity 89%, specificity 85%) compared with GPT-5.0 (sensitivity 67%, specificity 49%), largely driven by false-positive TR4 classifications among benign nodules. **Conclusions:** GPT-5.0 recognizes several high-level TI-RADS features but struggles with microcalcifications and tends to overestimate malignancy risk within a risk-stratification framework, limiting its standalone clinical use. Ultrasound-specific training and domain adaptation may enable meaningful adjunctive roles in thyroid nodule assessment.

## 1. Introduction

Thyroid nodules are highly prevalent in the general population, and ultrasound (US) is the primary imaging modality for their initial detection and characterization [1,2]. As a noninvasive, widely available technique, ultrasound plays a central role in risk stratification of these nodules, particularly in guiding fine-needle aspiration biopsy decisions [3]. However, thyroid ultrasound interpretation is inherently operator-dependent and susceptible to variation in experience, training, and subjective pattern recognition [4]. To address this variability, the American College of Radiology (ACR) introduced the Thyroid Imaging Reporting and Data System (TI-RADS), a structured scoring system designed to standardize feature evaluation and enhance diagnostic reproducibility. TI-RADS is now widely adopted in clinical practice and research, providing a consistent framework for assessing composition, echogenicity, shape, margin characteristics, and echogenic foci, each of which contributes to an overall estimate of malignancy risk [5,6,7].

Recent advances in artificial intelligence have introduced new possibilities for augmenting diagnostic imaging workflows. Deep learning-based image classification models have demonstrated promising results in thyroid nodule characterization; however, these systems typically require large annotated datasets, extensive preprocessing, and labor-intensive training pipelines [8]. In contrast, the emergence of large language models (LLMs) with multimodal capabilities—such as GPT-5.0—represents a fundamentally different paradigm. These models can process natural language, visual inputs, and complex contextual cues without domain-specific training, raising the question of whether they can interpret medical images using general reasoning capabilities alone [9]. Prior studies in thyroid imaging have mainly evaluated LLMs in text-based contexts, such as analyzing TI-RADS reports [10]. At the same time, a recent investigation explored the use of GPT-4 to assist decision-making in thyroid US using a chain-of-thought framework [11]. However, LLM performance on raw ultrasound images—an inherently noise-prone, operator-dependent modality—remains largely untested.

Thyroid ultrasound provides an ideal test case for LLM-based image interpretation, given the structured, rule-based nature of TI-RADS and the well-documented interobserver variability among radiologists [4,5]. If a multimodal LLM could reliably identify TI-RADS features directly from raw US images, it could enhance reproducibility, streamline triage workflows, and potentially reduce unnecessary biopsies. Yet, the subtle features required for accurate TI-RADS scoring—particularly echogenic foci and fine margin irregularities—pose substantial challenges for models that are not explicitly trained on ultrasound datasets.

To our knowledge, no previous study has systematically evaluated GPT-5.0’s performance in direct TI-RADS-based interpretation of thyroid ultrasound images using cytology-confirmed outcomes as the reference standard. This study does not aim to validate GPT-5.0 as a diagnostic tool or to equate TI-RADS categories with pathological diagnosis. Instead, it evaluates whether a general-purpose multimodal large language model can correctly apply a structured TI-RADS framework in nodules with clearly defined cytologic outcomes, providing insight into model behavior and failure modes under relatively low diagnostic ambiguity. By providing one of the first structured analyses of GPT-5.0 in thyroid ultrasound interpretation, this work highlights both the opportunities and current limitations of multimodal LLMs in ultrasound imaging.

## 2. Materials and Methods

### 2.1. Study Design and Ethical Approval

This retrospective, single-center study was conducted in accordance with the Declaration of Helsinki and was approved by the local Institutional Review Board (2025/108). Because the analysis used previously archived and anonymized ultrasound images and cytology results, the requirement for informed consent was waived. The study adhered to STROBE guidelines for observational research.

### 2.2. Patient Selection

A total of 100 consecutive patients who underwent thyroid ultrasound followed by fine-needle aspiration (FNA) cytology between January 2023 and December 2024 were evaluated. Patients were eligible for inclusion if longitudinal and transverse ultrasound images of diagnostic quality were available and if the corresponding FNA cytology result was definitive, classified as either Bethesda II (benign) or Bethesda V–VI (malignant). Indeterminate cytology categories (Bethesda III, IV), nondiagnostic or inadequate samples (Bethesda I), ultrasound examinations with severe artifacts or incomplete image acquisition, and cases lacking paired longitudinal–transverse images were excluded. Patients were retrospectively selected based on definitive cytology results (Bethesda II or V–VI). Indeterminate cytology categories (Bethesda III–IV), although clinically relevant, were excluded by design to allow assessment of model performance in a lower-ambiguity setting and to avoid confounding agreement analyses with intrinsic cytologic uncertainty. Because surgical histopathology was not systematically available in this cohort, FNA cytology served as the sole reference standard. Accordingly, all nodules were dichotomized as benign (Bethesda II) or malignant (Bethesda V–VI) for subsequent analyses. All DICOM images were anonymized and exported as high-resolution PNG/JPEG files to ensure uniformity during model evaluation.

### 2.3. Ultrasound Image Acquisition

Ultrasound examinations were performed using a high-frequency linear transducer (12–15 MHz) on a premium-grade system (Toshiba Aplio 500; Canon Medical Systems, Tokyo, Japan). Standard thyroid presets were used with optimized gain, dynamic range (60–70 dB), and focal zone placement. For each nodule, a static longitudinal and transverse image was obtained at its maximal diameter. No annotations, calipers, or Doppler overlays were included.

### 2.4. Radiologist Assessment

A board-certified radiologist with over 10 years of experience in thyroid imaging independently reviewed all images. The comparison with an expert radiologist was intentionally asymmetric and was not intended to benchmark human-level diagnostic performance. Instead, it was designed to contextualize GPT-5.0’s TI-RADS–based outputs relative to a clinical reference standard, enabling systematic analysis of model behavior, feature-level agreement, and failure modes. The radiologist was blinded to all clinical, demographic, and cytology results. For each nodule, TI-RADS features—composition, echogenicity, shape, margin, and echogenic foci—were scored using the 2017 ACR TI-RADS lexicon. A final TI-RADS category (TR1–TR5) was assigned according to the cumulative point-based system [5].

### 2.5. GPT-5.0 Image Assessment

A multimodal implementation of GPT-5.0 (OpenAI, 2025 release) was used to interpret the ultrasound images. The model was accessed via the OpenAI web-based user interface between January and February 2025. At the time of the study, no more granular public model identifier beyond “GPT-5.0” was provided by the developer.

All analyses were performed using static grayscale ultrasound images exported in high-resolution PNG/JPEG format. For each nodule, the longitudinal and transverse images were uploaded together in the same session, allowing the model to evaluate both views simultaneously, as a human reader would. Each case was assessed on a separate, fresh session with conversation memory disabled to prevent cross-case contamination or contextual carryover. No clinical information or metadata were provided. A standardized prompt was used for every image to ensure consistency: “*You are a radiology expert. Analyze this thyroid ultrasound image using the ACR TI-RADS system. Describe the composition, echogenicity, shape, margin, and echogenic foci. Then assign a TI-RADS category (TR1–TR5). Base your interpretation solely on the visual features seen in this image*.” Explicit TI-RADS definitions, point tables, or example cases were not included in the prompt; therefore, all categorizations relied on the model’s internal knowledge rather than externally supplied criteria. GPT-5.0 generated a single unified TI-RADS assessment for each nodule. To assess repeatability, the model re-evaluated 10 randomly selected cases under identical prompting conditions, yielding near-identical outputs in 9 of 10 cases.

### 2.6. TI-RADS Feature Scoring

All features were mapped to the point-based ACR TI-RADS scoring system to generate cumulative scores for final category assignment (TR1–TR5). Radiologist-based and GPT-5.0–based feature scores were recorded separately. For descriptive reporting, we also provided the proportion of nodules assigned to the most suspicious level within each feature category (e.g., solid composition, markedly hypoechoic echogenicity, taller-than-wide shape, irregular margins, and punctate echogenic foci).

### 2.7. Statistical Analysis

Statistical analysis was performed with SPSS version 26.0 (IBM Corp., Armonk, NY, USA). Agreement between GPT-5.0 and the radiologist was assessed using Cohen’s kappa (κ). Interpretation of κ followed Landis–Koch benchmarks.

Diagnostic performance—sensitivity, specificity, positive predictive value, and negative predictive value—was calculated using the cytology-based benign/malignant classification. For these analyses, TI-RADS categories TR4–TR5 were considered positive for malignancy, whereas TR2–TR3 were considered negative. Ninety-five percent confidence intervals (95% CIs) for sensitivity and specificity were calculated using the Wilson method. Additional analyses evaluated discordant cases and patterns of overclassification.

## 3. Result

### 3.1. Baseline Patient and Nodule Features

A total of 100 patients (mean age 49.8 ± 12.6 years; range 21–78 years) with 100 thyroid nodules were included. The cohort comprised 72 women (72%) and 28 men (28%). The median nodule size was 18 mm (IQR 12–26 mm). Nodules were in the right lobe in 56 cases, the left lobe in 38 cases, and the isthmus in 6 cases. According to the FNA-based reference standard, 27 nodules (27%) were classified as malignant, including 15 Bethesda V and 12 Bethesda VI lesions, while 73 nodules (73%) were classified as benign (Bethesda II) (Table 1). All nodules had both longitudinal and transverse images suitable for analysis.

### 3.2. Feature-Level Agreement Between GPT-5.0 and the Radiologist

Agreement across individual TI-RADS features varied considerably. GPT-5.0 demonstrated the highest consistency with the radiologist in assessing shape (κ = 0.70) and margin characteristics (κ = 0.68), indicating substantial agreement. Composition also showed a strong level of concordance (κ = 0.62), suggesting that GPT-5.0 could reliably identify whether a nodule was predominantly solid or mixed. In contrast, agreement for echogenicity was only moderate (κ = 0.48), reflecting more frequent discrepancies in distinguishing hypoechoic versus isoechoic or mildly hyperechoic nodules. The lowest agreement was observed for echogenic foci, with a poor kappa value (κ = 0.12). GPT-5.0 consistently underdetected punctate echogenic foci and microcalcifications, which contributed significantly to downstream category misclassifications.

In contrast, intraobserver analysis of the expert radiologist demonstrated consistently higher reliability across all individual TI-RADS features. Agreement was substantial for composition (κ = 0.75) and margin (κ = 0.76), moderate for echogenicity (κ = 0.57), and almost perfect for shape (κ = 0.88) and echogenic foci (κ = 0.91). These findings indicate that feature-level discrepancies observed in GPT-5.0 assessments exceed the intrinsic variability of expert human interpretation and primarily reflect model-specific limitations rather than reader inconsistency (Table 2).

**Table 2:** Agreement between the radiologist and GPT-5.0 for individual TI-RADS features.

TI-RADS Feature	Radiologist (%)	GPT-5.0 (%)	Cohen’s κ (95% CI)	Agreement Level	Radiologist Intraobserver Cohen’s κ	Agreement Level
***Composition***	64	60	0.62 (0.47–0.75)	Substantial	0.75 (0.63–0.85)	Substantial
***Echogenicity***	45	35	0.48 (0.32–0.63)	Moderate	0.57 (0.44–0.69)	Moderate
***Shape***	18	15	0.70 (0.55–0.82)	Substantial	0.88 (0.79–0.95)	Almost Perfect
***Margin***	22	25	0.68 (0.52–0.81)	Substantial	0.76 (0.64–0.86)	Substantial
***Echogenic foci***	27	3	0.12 (0.00–0.28)	Poor	0.91 (0.94–0.97)	Almost Perfect

### 3.3. TI-RADS Category Assignment

**Figure 1:**
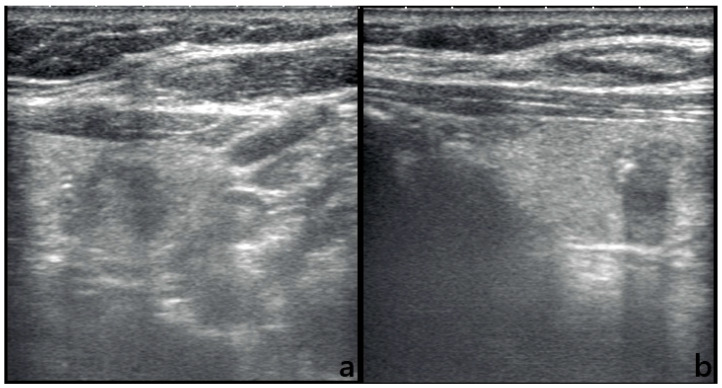
Ultrasound images of a thyroid nodule with discrepant ACR TI-RADS classifications between the radiologist and GPT-5.0. (**a**,**b**) show transverse and longitudinal ultrasound images of the same nodule. The radiologist classified this lesion as TI-RADS 5, highlighting its markedly hypoechoic appearance, irregular margin, and punctate echogenic foci suggestive of microcalcifications. In contrast, GPT-5.0 assigned the nodule a TI-RADS score of 4.

The radiologist most commonly classified nodules as TR1–TR2 (*n* = 30) and TR4 (*n* = 28), while GPT-5.0 tended to assign nodules to higher suspicion categories, particularly TR4 (*n* = 40) and TR5 (*n* = 22). This upward shift in categorization led to a moderate overall agreement rate of 58% (95% CI: 48–68%) between GPT-5.0 and the expert radiologist, with a corresponding Cohen’s κ of 0.31 (95% CI: 0.17–0.45) (Table 3). The majority of discordant cases involved GPT-5.0 assigning a higher TI-RADS category than the radiologist, reflecting the model’s tendency toward conservative, risk-averse classification. Importantly, these disagreements were not uniformly distributed across categories but clustered around intermediate-to-high suspicion transitions, with most discordant cases involving shifts from TR2 or TR3 to TR4 rather than extreme cross-category errors, such as assigning TR1 to TR5. An illustrative example of such discordance—a nodule classified as TI-RADS 5 by the radiologist but downgraded to TI-RADS 4 by GPT-5.0—is shown in Figure 1. Notably, several benign nodules with subtle echogenic artifacts or ill-defined margins were placed into the TR4–TR5 range by GPT-5.0 despite being scored lower by the expert reader.

In contrast, intraobserver analysis of the expert radiologist demonstrated substantially higher consistency in final TI-RADS category assignment, with an observed agreement of 80.8% (95% CI: 65–91%) and a Cohen’s κ of 0.74 (95% CI: 0.52–0.89) (Table 3). These findings indicate that the magnitude of disagreement observed between GPT-5.0 and the expert radiologist exceeds the intrinsic intraobserver variability of human interpretation and is therefore primarily attributable to model-specific limitations rather than reader inconsistency.

### 3.4. Outcome-Referenced Binary Classification Performance Against FNA Cytology

**Figure 2:**
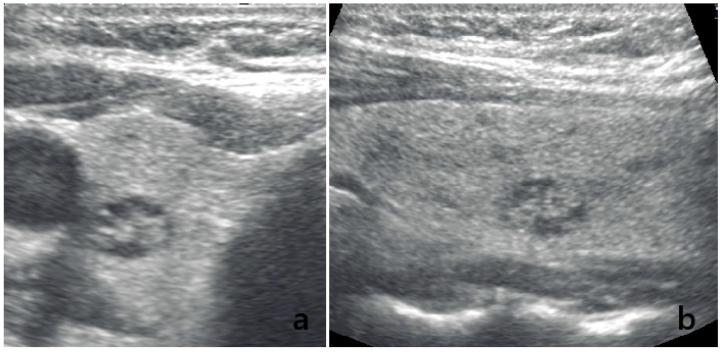
Ultrasound images of a benign thyroid nodule with discrepant ACR TI-RADS classifications between the radiologist and GPT-5.0. (**a**,**b**) show two orthogonal ultrasound views of the same nodule. The radiologist classified the lesion as TI-RADS 2, noting its isoechoic appearance, smooth margins, and absence of suspicious sonographic features, all of which are consistent with a benign profile. In contrast, the GPT-5.0 model assigned the nodule a TI-RADS 4 score.

For descriptive and contextual purposes, FNA cytology was used as an outcome reference, and TI-RADS categories TR4–TR5 were considered positive for malignancy (TR2–TR3 negative). Under this binary framework, the radiologist achieved a sensitivity of 88.9% (24/27; 95% CI, 71.9–96.1), a specificity of 84.9% (62/73; 95% CI, 75.0–91.4). In contrast, GPT-5.0 demonstrated lower outcome-referenced binary classification performance, with a sensitivity of 66.7% (18/27; 95% CI, 47.8–81.4) and a specificity of 49.3% (36/73; 95% CI, 38.2–60.5) (Table 4). The reduced specificity reflected the model’s strong tendency to overcall malignancy risk, particularly in benign nodules misinterpreted as TR4 or TR5 due to false identification of suspicious features. A representative benign nodule that was accurately classified as TI-RADS 2 by the radiologist but overclassified as TI-RADS 4 by GPT-5.0 is shown in Figure 2. This pattern resulted in a substantial increase in false-positive classifications. When viewed within a binary decision framework (TR4–TR5 vs. TR1–TR3), this misclassification pattern demonstrates that the reduced specificity of GPT-5.0 is driven predominantly by false-positive assignments arising from benign nodules shifted into the TR4 category, rather than by widespread category confusion. These binary outcome-referenced metrics are reported to illustrate patterns of risk redistribution and false-positive classification rather than to imply diagnostic validation of TI-RADS or GPT-5.0.

### 3.5. Malignancy Rates Across TI-RADS Categories

For the radiologist, malignancy rates increased progressively across TI-RADS categories (Table 2), consistent with expected TI-RADS behavior: 3% in TR2, 8% in TR3, 35% in TR4, and 85% in TR5. GPT-5.0 demonstrated the same directional trend; however, malignancy proportions in intermediate categories were inflated (0% in TR2, 10% in TR3, and 30% in TR4), reflecting the model’s tendency to up-classify benign nodules into higher suspicion categories. As a result, the apparent increase in malignancy within GPT-5.0’s TR3–TR4 assignments represents risk redistribution rather than improved detection performance. In a review of discordant cases, most errors were attributable to the overinterpretation of margin irregularities and shape-related features, whereas echogenic foci were more frequently underdetected rather than overcalled.

## 4. Discussion

This study provides one of the first structured evaluations of a large language model (LLM), GPT-5.0, for the direct interpretation of raw thyroid ultrasound images using the ACR TI-RADS lexicon, and is among the earliest works to benchmark pixel-level TI-RADS feature recognition against expert radiologist interpretation using cytology-confirmed outcomes. Our findings demonstrate that GPT-5.0 can identify several high-level morphological features—particularly shape, margins, and overall composition—with meaningful consistency. These characteristics tend to be visually dominant, geometrically stable, and less dependent on subtle echogenic patterns, which likely explains the model’s substantial agreement with the expert radiologist. Similar observations have been reported in prior AI-based thyroid imaging studies, where global nodule architecture is more reliably detected than delicate internal structures [8,12,13].

In contrast, feature-level performance declined sharply for echogenic foci. Microcalcifications remain among the most powerful sonographic indicators of malignancy, yet they are also among the most technically challenging to identify consistently [14,15]. Even domain-specific deep learning systems trained on large thyroid ultrasound datasets frequently misclassify microcalcifications due to speckle noise, acoustic interference, and small pixel density [16,17,18]. The poor agreement for echogenic foci in our study, therefore, highlights a fundamental limitation of general-purpose multimodal LLMs: their lack of ultrasound-optimized visual encoders. This is consistent with broader research showing that current vision–language models are less sensitive to high-frequency grayscale patterns than conventional convolutional architectures [19].

Our findings also extend the growing literature on LLM applications in thyroid imaging. Most existing studies have evaluated LLMs using text-based inputs. Sievert et al. demonstrated that ChatGPT’s TI-RADS performance was dependent on structured report descriptors rather than raw imaging features [10]. At the same time, Wakonig et al. showed similar limitations for GPT-4.0 in report-based TI-RADS categorization [20]. Köroğlu et al. assessed ChatGPT for thyroid nodule management pathways rather than imaging interpretation [21]. Wang et al. explored GPT-4 for clinical reasoning based on descriptive text instead of image analysis [11]. Cabezas et al. performed one of the earliest attempts at image-based risk estimation using GPT-4, but reported inconsistent and error-prone feature identification [22]. In contrast to these prior works, our study directly evaluates pixel-level TI-RADS feature recognition by an advanced multimodal LLM, using cytology-confirmed outcomes as the reference standard.

A notable pattern in our analysis was GPT-5.0’s systematic tendency to overclassify nodules into higher TI-RADS categories, especially TR4–TR5. Similar “safety-biased overcalling” has been documented in several deep learning studies, particularly when models struggle to differentiate microcalcifications from benign interfaces or colloid clusters [8,16,23]. This tendency led to inflated malignancy rates in intermediate TI-RADS categories and significantly reduced specificity and overall accuracy in our cohort. Although such conservative bias may appear clinically cautious, it contradicts a principal goal of TI-RADS: reducing unnecessary biopsies and preventing overdiagnosis [5,7,19]. However, despite these partial strengths, GPT-5.0’s overall agreement with the cytology reference standard remained poor, underscoring that the model should not be used as a stand-alone diagnostic reader in thyroid nodule assessment. Accordingly, performance differences between GPT-5.0 and the expert radiologist should not be interpreted as evidence of model inadequacy, but rather as informative indicators of the current limitations and application boundaries of general-purpose multimodal language models in thyroid ultrasound interpretation.

Significantly, this systematic overclassification was not driven by over-detection of echogenic foci. Although microcalcifications represent a high-risk TI-RADS feature, GPT-5.0 frequently failed to identify punctate echogenic foci. Instead, the upward shift in TI-RADS categorization resulted from the cumulative effect of other suspicious features—particularly shape, margin, and echogenicity—which exert dominant influence within the ACR TI-RADS framework. This distinction underscores the importance of separating feature-level detection performance from category-level risk stratification when interpreting model behavior.

Importantly, intraobserver agreement for individual TI-RADS features ranged from moderate to almost perfect, whereas agreement for final TI-RADS category assignment was substantially higher for repeat expert readings than for GPT-5.0–radiologist comparisons. This discrepancy reflects the cumulative, threshold-based nature of the TI-RADS scoring system, in which slight variations in one or two features can shift nodules between adjacent categories (e.g., TR3 versus TR4). The markedly higher intraobserver agreement observed at both the feature level (Table 2) and the category level (Table 3) supports the internal consistency of expert human interpretation. It indicates that category-level disagreements primarily reflect model-specific limitations rather than reader variability.

Despite these limitations, GPT-5.0 exhibited strengths with meaningful potential for real-world applications. Its substantial agreement for shape and margin characteristics suggests that LLM-based systems may excel at detecting global geometric outlines and larger-scale morphological patterns—an area where manual TI-RADS assessments are known to suffer from interobserver variability [4,24,25]. These capabilities support plausible roles for GPT-5.0 in nodule triage, automated pre-screening, standardized structured reporting, and radiology education. Prior research has shown that LLMs can support clinical decision-making, enhance interpretability, and improve trainee consistency in radiology settings [9,26,27], further highlighting their usefulness as adjunctive tools rather than standalone diagnostic readers.

Several limitations warrant discussion. First, GPT-5.0 was evaluated exclusively using static grayscale images. Multiparametric ultrasound, including elastography and Doppler evaluation, has demonstrated improved diagnostic performance in previous studies [28,29], and such inputs may benefit future multimodal architectures. Second, GPT-5.0 was not fine-tuned on ultrasound-specific datasets; thus, its performance represents general-purpose multimodal reasoning rather than domain-optimized capability. The internal image preprocessing steps applied by GPT-5.0, including potential resizing, downsampling, or compression after image upload, are not disclosed by the developer and therefore constitute an inherent limitation of this study, particularly for detecting fine image details such as microcalcifications. Advances in foundation model research demonstrate that task-specific fine-tuning or adapter-based modules substantially improve accuracy and generalization in medical imaging applications [30]. Third, this was a retrospective, single-center study, which limits generalizability. Larger multicenter cohorts with more heterogeneous imaging conditions would strengthen external validity. Fourth, a selection bias is inherent in the study design: only nodules that underwent FNA cytology were included, which likely enriched the cohort for higher-risk lesions and may have overestimated the prevalence of malignancy compared with unselected ultrasound populations. This high-risk sampling differs from typical TI-RADS screening cohorts, where malignancy rates are substantially lower. Because surgical histopathology was not systematically available, FNA cytology served as the sole reference standard in this study. Although widely used in clinical practice, FNA is subject to sampling variability and potential misclassification, which may have influenced diagnostic performance estimates and should be considered when interpreting the results. Finally, although repeatability testing demonstrated stable outputs, broader reproducibility analyses—including perturbation robustness and prompt-sensitivity testing—will be essential as multimodal LLMs continue to evolve. Moreover, because indeterminate nodules represent the primary clinical target of TI-RADS, their exclusion constitutes a significant limitation. Accordingly, the findings should not be extrapolated to clinical scenarios involving Bethesda III–IV nodules or real-world TI-RADS–guided biopsy decision pathways. In this context, sensitivity and specificity values in this study should be interpreted as descriptive indicators of model behavior under a binary outcome framework, not as measures of diagnostic accuracy.

## 5. Conclusions

In conclusion, GPT-5.0 demonstrates encouraging capabilities in identifying high-level TI-RADS features directly from thyroid ultrasound images; however, it currently lacks the fine-grained visual sensitivity required for reliable clinical decision-making. Its strong performance in recognizing global morphological patterns suggests potential utility in triage workflows, structured reporting, and educational applications. However, substantial refinement—including ultrasound-specific training, optimized visual encoders, and prospective multicenter validation—is necessary before multimodal LLMs can be integrated safely into clinical thyroid imaging pathways. Continued progress in vision–language architectures will determine whether next-generation LLMs can ultimately meet the precision required for ultrasound-based risk stratification.

## Figures and Tables

**Table 1:** Patient and Nodule Characteristics.

Characteristic	Value
*Number of patients*	100
*Age, mean ± SD (range)*	49.8 ± 12.6 (21–78)
*Sex*	
• *Female*	72 (72%)
• *Male*	28 (28%)
*Nodule size, median (IQR)*	18 mm (12–26 mm)
*Nodule location*	
• *Right thyroid lobe*	56 (56%)
• *Left thyroid lobe*	38 (38%)
• *Isthmus*	6 (6%)
*FNA Cytology*	
• *Malignant nodules (Bethesda V–VI)*	27 (27%)
• *Benign nodules (Bethesda II)*	73 (73%)

**Table 3:** Distribution of TI-RADS categories and malignancy rates for the radiologist and GPT-5.0.

TI-RADS Category	Radiologist (*n*)	Radiologist Malignancy (%)	GPT-5.0 (*n*)	GPT-5.0 Malignancy (%)
***TR2***	30	3%	18	0%
***TR3***	25	8%	20	10%
***TR4***	28	35%	40	30%
***TR5***	17	85%	22	60%
***Radiologist vs. GPT-5.0***				58% (κ = 0.31)
***Radiologist intraobserver***	—	—	—	80% (κ = 0.74)

**Table 4:** Diagnostic performance of the radiologist and GPT-5.0 using FNA cytology as the reference standard.

Method	Sensitivity (95% CI)	Specificity (95% CI)	Cohen’s κ
***Radiologist***	88.9% (71.9–96.1)	84.9% (75.0–91.4)	0.52
***GPT-5.0***	66.7% (47.8–81.4)	49.3% (38.2–60.5)	0.18

## Data Availability

The data presented in this study are available from the corresponding author on reasonable request. The data are not publicly available due to privacy reasons.
